# Distinct Antiviral Properties of Two Different Bacterial Lysates

**DOI:** 10.1155/2021/8826645

**Published:** 2021-02-04

**Authors:** Michael Roth, Hanif J. Khameneh, Lei Fang, Michael Tamm, Giovanni A. Rossi

**Affiliations:** ^1^Pulmonary Cell Research, DBM University Basel and Pneumology Clinic, University Hospital Basel, Basel, Switzerland; ^2^Institute for Research in Biomedicine, Faculty of Biomedical Sciences, Università Della Svizzera Italiana, Bellinzona, Switzerland; ^3^Department of Pediatrics, Pulmonology and Allergy Units, IRCCS Istituto Giannina Gaslini, Genoa, Italy

## Abstract

Oral bacterial lysates (OBLs) can reduce the frequency and severity of recurrent respiratory tract infections in children from viral and bacterial origins. OBL-induced early innate immune reaction was already shown, but the specific features of different OBLs have never been studied and compared. A study was conducted to assess in vitro the protective effects on rhinovirus- (RV-) infected human bronchial epithelial cells (BECs) of two slightly different OBLs: OM-85 and Pulmonarom. Furthermore, since immune cells represent the key arm for antiviral defence, the capacity of these OBLs to induce selected cytokine production in mouse bone marrow-derived DCs (BMDCs) was also evaluated. Although different OBLs may share some mechanisms to protect host cells from virus infection, some product-specific antimicrobial activities were observed on RV-infected human BECs and mouse BMDCs. These results are consistent with a product-specific response possibly triggered by different pathogen-associated molecular patterns (PAMPs) contained in OBLs.

## 1. Introduction

Viral infections of the upper and lower respiratory tract are highly prevalent in children [1–3] and present a major risk factor for the development of persistent bronchial hyperresponsiveness in early childhood. Viral infections are the main cause of recurrent wheezing and asthma exacerbations at all ages [4, 5]. Two recent studies provided evidence that preventive treatment with a bacterial lysate (OM-85) in children at risk of severe lower respiratory tract infections significantly reduced the frequency of viral infections [6, 7]. However, the cell biological mechanism underlying this protective effect of OM-85 remains incompletely understood.

Airway epithelial cells are the primary entry site for viral infections and control inflammation and immune response [8]. Airway epithelial cells express pattern recognition receptors (PRRs) that detect environmental stimuli, such as virus or bacterial particles, and promote the release of endogenous danger signals, defensive cytokines, and antiviral molecules [8]. Rhinovirus (RV) is the most common cause of recurrent wheezing and the development of childhood asthma [9]. Bronchial epithelial cells (BECs) from children with wheezing and asthma had a reduced immune response to RV infection when compared to BECs from children without asthma [10]. Epithelial cells obtained by bronchial brushing from asthmatic children had a reduced IFN-*β* production that was associated with inhibition of host cell apoptosis, increased production of inflammatory cytokines, increased virus replication, and impaired wound repair capacity [11].

Deficiency of the immune responses to common virus infections is not related to allergic sensitization, since deficient antiviral immune responses were detectable in bronchial biopsies from asthmatic children, irrespective of their atopic status [12]. In these children, low IFN-*β* levels inversely correlated with RV load, airway Th2 immunopathologic profile (eosinophilia and IL-4 positivity), and epithelium damage [12]. The epidemiological observation that early-in-life exposure to microbes may increase the efficiency of the immune responses and prevent the onset of wheezing and asthma through “nonspecific immunomodulation” suggests that bacterial lysates might enhance the human natural defence system [13].

A systematic review showed that bacterial lysates can effectively prevent recurrent respiratory tract infections and reduce the incidence, severity, and duration of symptoms [14]. Bacterial lysates are thought to activate immune effector cells through the interaction of their conserved pathogen-associated molecular patterns (PAMPs) with toll-like receptors (TLRs) expressed by host cells [15]. TLR2 plays a major role in mediating and modulating antiviral activities against RV infection of airway epithelium but TLR7/8 are also involved [16, 17]. Another study showed that an oral bacterial lysate (OBL), OM-85, promoted BEC defence in response to RV-16 infection by modulating the expression of cell surface molecules and stimulating the expression of virus interacting C1q-R [18] and the antimicrobial peptide *β*-defensin [19]. However, it is not known to what extent these antiviral properties are common to different OBLs.

Therefore, an in vitro study was designed to assess and compare the protective effects induced on RV-16-infected BEC by two OBLs produced from the same bacterial genera but slightly different in bacterial strain composition and manufacturing process. An established, immortalized human bronchial epithelial cell line (hBEAS-2B) and three primary BEC cultures were first pretreated with either OM-85 or Pulmonarom and then infected with a commercially available RV-16 strain for up to 3 days. The effects of the two OBLs on BEC were assessed testing the following parameters: infection rate, BEC survival, expression of ICAM-1, and secretion of *β*-defensin-1 and IFN-*β*.

Considering the importance of immune cells and gut-lung axis in OBL lung protection against viral infections [13, 14, 20], it was also decided to study OBL-mediated cytokine induction by immune cells. To this end, we measured the production of the important immune mediators IFN-*β*, TNF-*α*, and IL-6 by mouse BMDCs, which are a commonly used in vitro model of innate immune cells [21, 22].

## 2. Materials and Methods

### 2.1. Human Cells

A stabilized, virus-transformed human bronchial epithelial cell line, BEAS-2B (ATCC, Cat# CRL-9609, Manassas, USA), was grown in 1 : 1 RPMI1640 and CnT-PR-A, supplemented with 10% fetal calf serum, 20 mM HEPES, and 10 mM sodium pyruvate BRL (Thermo Fisher Scientific, Reinach, Switzerland).

### 2.2. Primary Human Bronchial Epithelial Cells (BECs)

Three primary BEC lines were isolated from bronchial tissue samples obtained from cancer-control patients. BECs were grown in CnT-PR-A medium (CellnTec, Bern, Switzerland). All the experiments were performed once with each primary BEC culture and in quadruplicate in the BEAS-2B cells.

### 2.3. Cell Characterisation

Primary BECs were characterised by positive staining of E-Cadherin (Abcam 15148, Abcam, Cambridge, UK), pan-cytokeratin (sc-8018, Santa Cruz Bio technology, Santa Cruz, CA, USA), or cytokeratin-14 (Abcam 9220) and by negative staining for fibronectin (Abcam 23751) as described earlier [19]. In brief, cells were grown on 8-well chamber slides (Sarstedt, Sevelen, Switzerland) until 80% confluence and fixed for 2 × 5 minutes in 4% formalin. Cells were permeabilised for 5 minutes in PBS containing 0.1% TWEEN-20 and 0.05% Triton-X100 before being washed 3x with PBS-T (PBS + 0.1% Tween-20), and unspecific binding was blocked by 30-minute incubation in 2% skim milk in PBS-T and overnight incubation with one primary antibody. The slides were then washed 3x with PBS and incubated for 30 minutes at room temperature with a fluorescence labelled antibody specific for the species of the primary antibody (anti-mouse cat# R37114, anti-rabbit cat# R37119, Thermo Fisher). Slides were washed 3x with PBS before being monitored for fluorescence on an EVOS live-cell imaging station (Thermo Fisher).

### 2.4. Generation and Stimulation of Mouse Bone Marrow-Derived Dendritic Cells (BMDCs)

Bone marrow cells were extracted from femurs and tibias of 6–10-week-old wild-type C57/BL6 mice by flushing the bones with ice-cold PBS. BM cells were then plated on Petri dishes at a density of 1 × 106 cells/mL and cultured in Iscove's Modified Dulbecco's Medium (IMDM) with GlutaMax supplemented with 10% Fetal bovine serum (FBS), 1% pen-strep antibiotics, 2-mercaptoethanol, and HEPES (all from Gibco), in presence of 20 ng/mL recombinant mouse granulocyte-macrophage colony-stimulating factor (GM-CSF) (Peprotech). The culture was replenished with 5 mL of fresh medium 3 days later and, on day 7, floating cells were harvested by gentle agitation, spanned down, and resuspended in the medium for further applications. BMDCs were plated in a 96-well tissue culture plate at a density of 1.5 × 105 cells/well and stimulated for 16 hr with the indicated dilutions of OM-85 or Pulmonarom. The experiment was performed three times.

### 2.5. OM-85 and Pulmonarom

**Figure 1:**
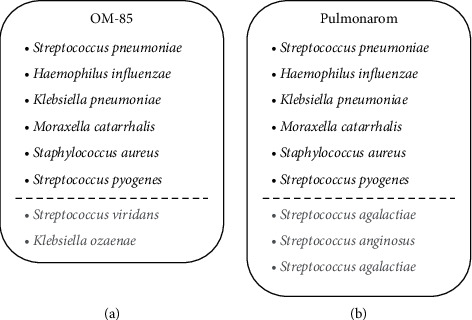
The composition of the two oral bacterial lysates. The composition of OM-85 differs from Pulmonarom. OM-85 contains *Streptococcus viridans* and *Klebsiella ozaenae*, instead of *Streptococcus agalactiae*, *Streptococcus dysgalactiae*, and *Streptococcus anginosus*.

The liquid form of OM-85 (drug intermediate) was kindly provided by OM Pharma SA (1217 Meyrin 1, Switzerland). The liquid form of Pulmonarom (drug product) was purchased as a commercial product. As no concentration of API (active principle ingredient) was known for Pulmonarom, serial volume dilutions were used to assess the difference in OBL-induced cytokine release. Both compounds consist of lysates from a range of similar bacteria as shown in Figure 1.

### 2.6. RV-16 Protein Immune Fluorescence

The RV-16 strain used had been described earlier, and the same RV-16 stock was used [19]. Based on preliminary experiments, epithelial cells were infected with 0.1, 0.5, or 1 MOI (multiplicity of infection) of RV-16 for up to 3 days. The infection rate was determined by immunofluorescence staining using an anti-RV-16 antibody (cat# 18758, QED-Bioscience Inc. San Diego, USA) as described earlier [19]. In brief, epithelial cells were seeded into 8-well chamber slides (Sarstedt) and allowed to adhere overnight. The cells were then treated for 24 hours with OM-85 or Pulmonarom before being infected with 0.1, 0.5, and 1.0 MOI of RV-16 for up to 72 hours. The cells were fixed in 4% formalin (in PBS, 5 min), washed 2x with PBS, and permeabilised by 0.05% Triton X-100 in PBS (15 min). After blocking with 2% bovine serum albumin (30 min), cells were incubated overnight (4°C) with anti-RV-16 antibody (1 : 100 dilution). Following 3x washes (PBS), the slides were incubated with a second FITC labelled antimouse antibody (1 hour, room temperature) and then washed 3x with PBS. The number of RV16 positive cells was counted by immunofluorescence microscopy (EVOS FLoid cell imaging station, Thermo Fisher Scientific).

### 2.7. Host Cell Survival

The survival of RV-16-infected BEC was determined in 80% subconfluent cells over 48 hours by immunofluorescence microscopy (EVOS Floid cell imaging station, Thermo Fisher Scientific) using a two-colour dual-parameter cell viability assay reagent (cat# L3224, Thermo Fisher Scientific). BECs were seeded (104 cells/cm^2^) and allowed to adhere overnight in the growth medium. The cells were then incubated with either OM-85 or Pulmonarom for 24 hours before being infected with 0.1, 0.5, and 1.0 MOI of RV-16 for up to 48 hours. The live-cell reagent was added for 30 minutes at 37°C, and the cells were monitored by an EVOS microscope. The number of cells was counted in an area of 100 × 100 *μ*m, which was defined as 100%. The number of green cells was counted in the same area, which indicates dead cells. The percentage of dead cells was determined in three different areas of the same slide as described earlier [19].

### 2.8. ELISAs

Secreted cytokines were detected by specific ELISA in cell culture supernatants, which were collected: (i) 24 hours after the addition of OM-85 or Pulmonarom, (ii) before RV-16 infection (controls), or (iii) 24 hours after RV-16 infection. The following commercial ELISA kits were obtained for *β*-defensin-1 (Antibodies online: beta-defensin 1 CLIA Kit : ABIN6202097), IFN-*β* (R&D System: Human IFN-beta Quantikine ELISA Kit : DIFNB0), and IFN-*γ* (R&D System: DY285). In mouse BMDC culture supernatants, cytokine release was measured by ELISA kits according to the manufacturer's instructions. IL-6 and TNF-*α* kits were from Biolegend and IFN-*β* from PBL Assay Science. The concentration of the cytokines was illustrated as mean+/−SEM of *n* = 3 technical replicates. The expression of ICAM-1 was detected by a commercial ELISA specific for the detection of ICAM-1 in cell extracts (ICAM-1 Kit : ABIN414384, Antibodies Online, Aachen, Germany). Cells were lysed and the protein content was determined by BCA (BCA protein quantitation kit: ABIN593356, Antibodies Online). An equal amount of total protein (50 *μ*g) was used for each sample, the ICAM-1 ELISA was performed according to the instructions of the distributor, and ICAM concentrations were determined by an ELISA reader (BioRad, Basel, Switzerland).

### 2.9. Statistics

The null hypothesis was that there is no effect of either OM-85 or Pulmonarom RV-16 infection or cell response or cytokine release. In epithelial cell experiments, statistics were calculated by ANOVA for dilution-dependent effects, followed by Student's *t*-test (paired, two-sided), and subsequent Wilcoxon test when applicable. *P* values <0.05 were considered as significant.

## 3. Results

### 3.1. RV Infection and RV-16-Infected BEC Survival

**Figure 2:**
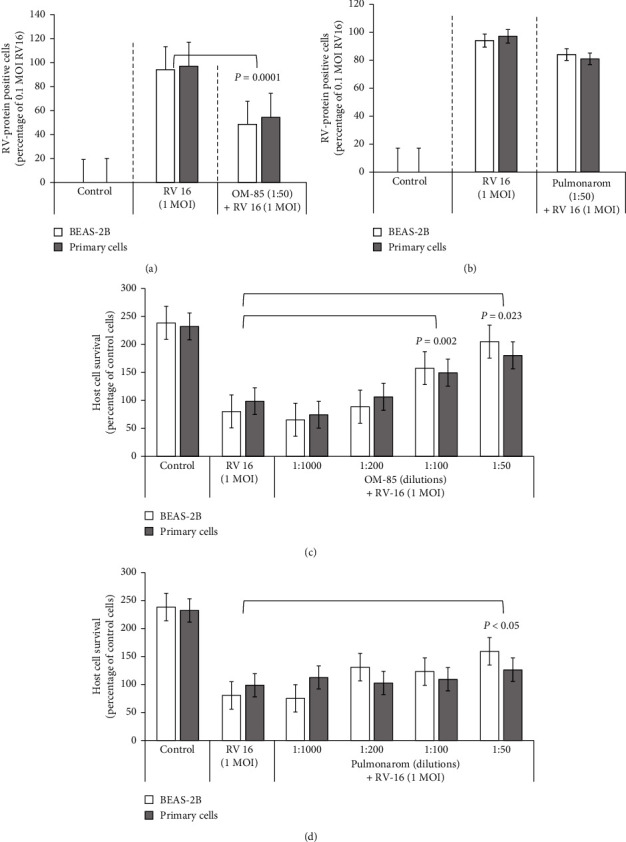
RV infection and RV-infected cells survival rates. The ratio of RV-16 positive BEAS-2B cells and primary human bronchial primary epithelial cells infected by 1.0 MOI RV-16 and pretreated with OM-85 (a) or Pulmonarom (b). Improved cell survival in 1.0 MOI RV-16-infected cultures pretreated with OM-85 (c) or Pulmonarom (d) at different dilutions (1 : 1000, 1 : 200. 1 : 100, 1 : 50). ICAM-1 expression and antiviral cytokine production.

The proportion of cells infected by RV-16 (≈100%) was not affected by the exposure to increasing RV-16 MOI. RV-16 infection was significantly reduced in cells pretreated with the highest dilution of OM-85 (*P*=0.0001) (Figure 2(a)). In contrast, RV-16 infection was not significantly reduced in Pulmonarom-pretreated cells (Figure 2(b)). Cell survival after RV-16 infection was MOI-dependent and decreased inversely with increasing RV-16 MOI (not shown). Both OBLs were able to improve cell survival of RV-16-infected BECs in a dilution-dependent manner. This protective effect was significant for the two highest OM-85 dilutions (Figure 2(c); *P*=0.002 and 0.023, respectively) and the highest Pulmonarom dilution (*P*=0.05) (Figure 2(d)). There were no significant differences comparing primary BEC to BEAS-2B cells.

**Figure 3:**
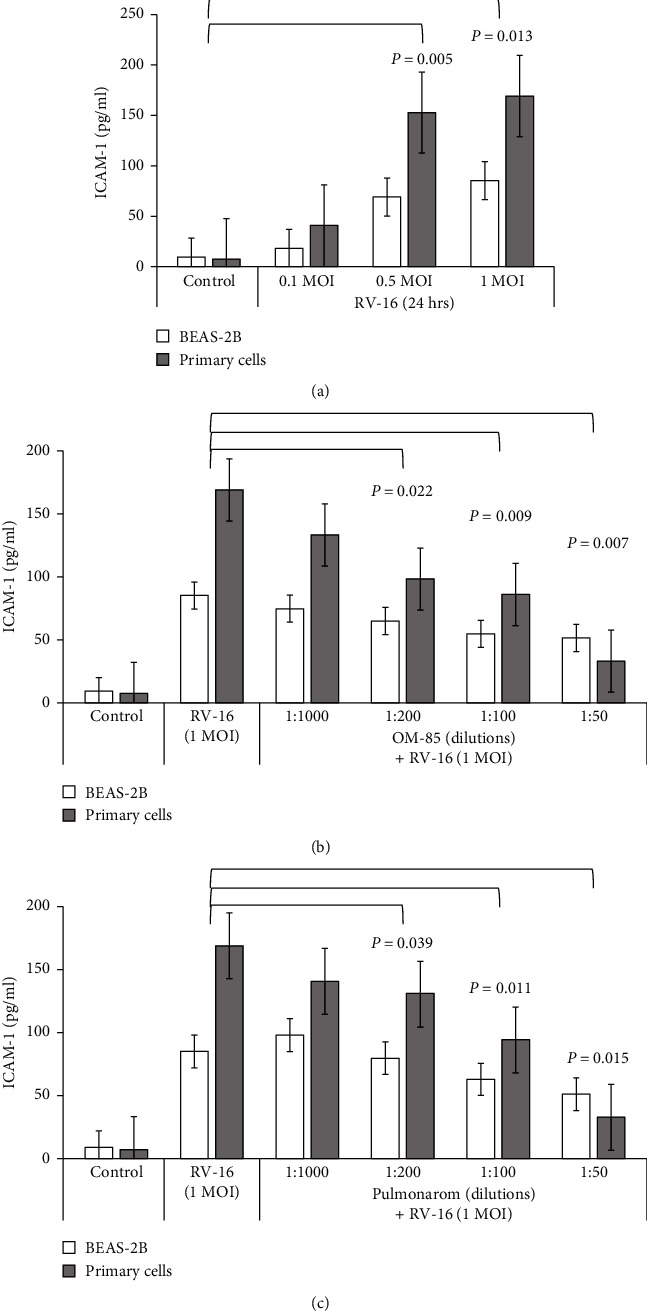
ICAM-1 expression. (a) ICAM-1 expression by BEAS-2B and primary cells infected by 1.0, 0.5, or 1.0 MOI RV-16. (b) The concentration-dependent effect of OM-85. (c) Pulmonarom on ICAM-1 expression by BEAS-2B and primary cells.

RV-16 infection upregulated the expression of ICAM-1 in a MOI-dependent manner (Figure 3(a)). This effect was significantly reduced in cells preincubated with OM-85 and Pulmonarom, at all the dilution tested (Figures 3(b) and 3(c)).

**Figure 4:**
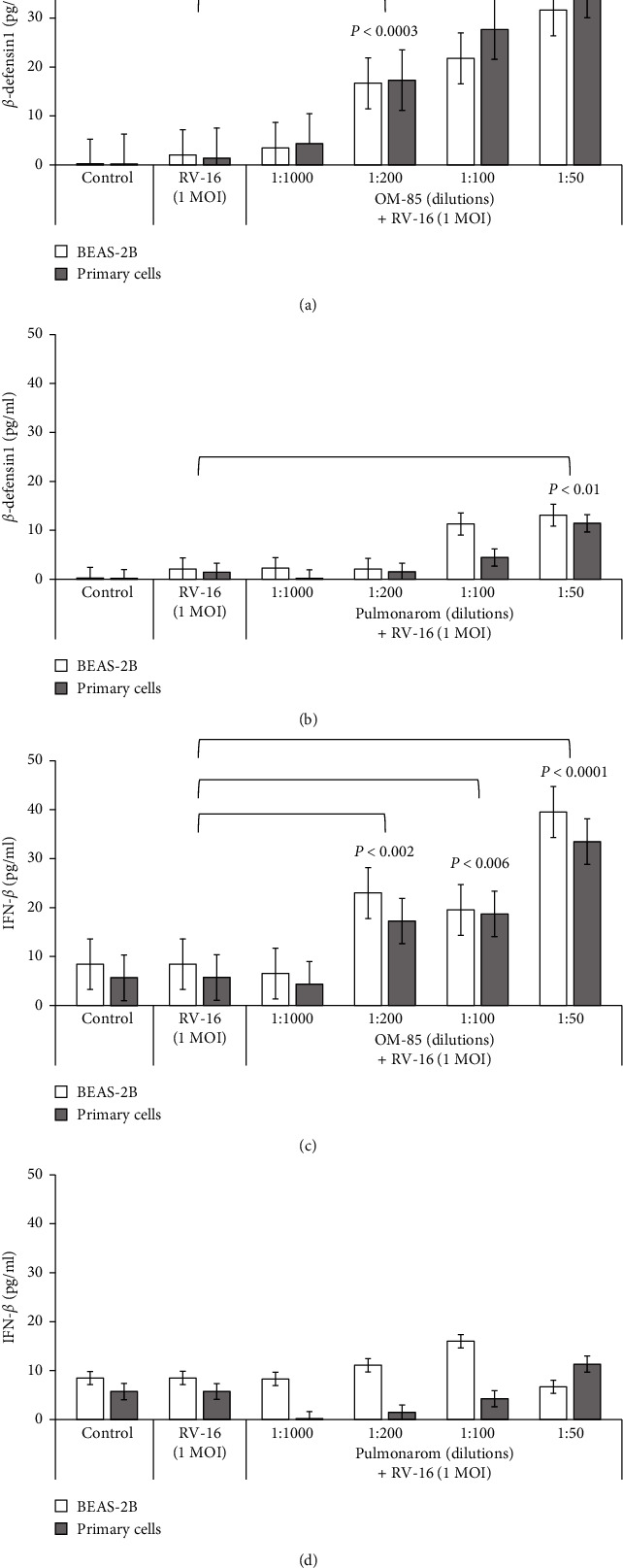
Modification of *β*-defensin-1 and IFN-*β* by bacterial lysates. *β*-defensin-1 release by BEAS-2B and primary cells infected with 1.0 MOI RV-16 after pretreatment with (a) OM-85 or (b) Pulmonarom at different concentrations after 24 hours. IFN-*β* release by BEAS-2B and primary cells infected by 1.0 MOI RV-16 and pretreated with increasing concentrations of either (c) OM-85 or (d) Pulmonarom.

The secretion of *β*-defensin-1 was upregulated by OM-85 and Pulmonarom in epithelial cells in a dilution-dependent manner (Figures 4(a) and 4(b)). The stimulating effect of OM-85 became significant at dilutions <1 : 200 (Figure 4(a)), whilst the stimulating effect of Pulmonarom did achieve significance only at dilutions <1 : 50 (Figure 4(b)). There was no difference comparing BEAS-2B cells and BEC. Only OM-85 increased the secretion of IFN-*β* in a dilution-dependent manner, with no significant difference comparing BEAS-2B cells to primary BEC (Figure 4(c)). The stimulatory effect of OM-85 became significant at dilution of <1 : 200/ml, whilst Pulmonarom did not achieve a significant effect at any dilution tested (Figure 4(d)).

### 3.2. Cytokine Production by BMDCs

**Figure 5:**
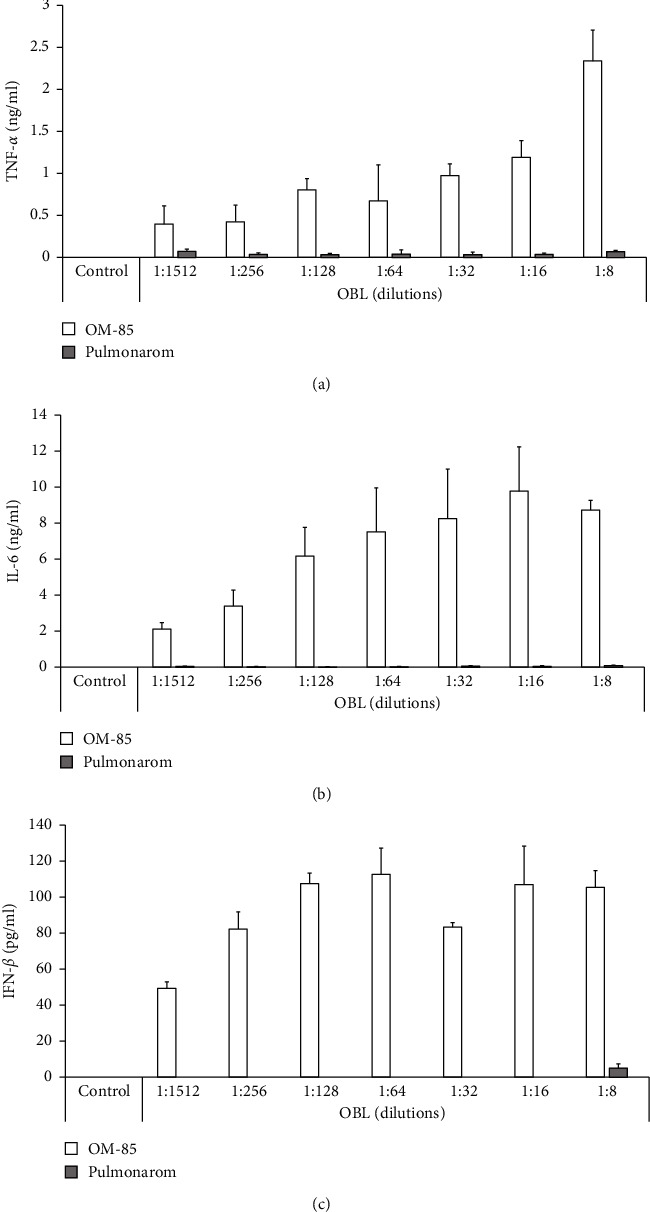
Bacterial lysate's effect on in vivo secretion of TNF-*α*, IL-6, and IFN-*β* by dendritic cells. The concentration-dependent effect of pretreatment with OM-85 or Pulmonarom on the secretion of (a) TNF-*α*, (b) IL-6, and (c) IFN-*β* by mouse bone marrow-derived dendritic cells.

Treatment of murine BMDCs with OM-85, but not with Pulmonarom, triggered the release of TNF-*α* and IL-6 and in a dose-dependent manner (Figures 5(a) and 5(b)). Furthermore, OM-85 induced the secretion of antiviral type I IFN-*β* by BMDCs, corroborating earlier results from human epithelial cells (Figure 5(c)).

## 4. Discussion

This study compared the in vitro antiviral effect and immune induction of two similar OBLs. To compare the antiviral activity induced by both liquid OBLs, we decided not to use the OM-85 commercial product formulated as a capsule. Instead, we used its corresponding soluble form prior to lyophilisation, the OM-85 liquid form. It is the drug intermediate of the final drug product and contains a pure active principle ingredient (API) as for Pulmonarom. Accordingly, both OBLs contain surface-derived soluble membrane microbial contents, so-called pathogen-associated molecular patterns (PAMPs), both from five bacterial genera, including *Streptococcus pneumoniae*, *Haemophilus influenzae*, *Klebsiella pneumoniae*, *Moraxella catarrhalis*, *Staphylococcus aureus*, *Streptococcus pyogenes*, *Streptococcus viridans*, and *Klebsiella ozaenae* as depicted in Figure 1(a). Pulmonarom is a commercial active liquid form and differs from OM-85 in three strains only as shown in Figure 1(b). For the antiviral defence, RV-16 infection was used in two bronchial epithelial cell types (primary and immortalized cell lines).

The results show that OM-85 was more effective in reducing RV-16-infection and ICAM expression as well as in inducing the release of *β*-defensin and IFN-*β*. OM-85 was also more efficient to improve host cell survival. The significant difference in the RV-16-induced changes of ICAM-1 expression comparing BEAS-2B cells to BEC suggests that immortalized cell lines may not fully reflect the response of normal BEC. Data on murine BMDCs confirmed that OM-85 induced cytokine responses, including antiviral IFN-*β* production, whilst no measurable amounts of IFN-*β*, IL-6, and TNF-*α* were detected upon treating cells with Pulmonarom.

Epidemiologic and clinical studies provided evidence that RV infection is the leading cause of respiratory morbidity and an important cause of airway injury and remodelling, which may lead to persistent bronchial hyperactivity, wheezing exacerbations, and possibly asthma [23, 24].

In the scope of asthma and respiratory infections, it is generally accepted that preventive measures are based on strengthening the host's immune system, thus, increasing the natural response to pathogens. In this context, it was reported that OM-85 positively affects the physiology and function of respiratory epithelial cells and improves their host defence mechanisms [25]. In two clinical studies, OM-85 prevented the reoccurrence of respiratory drug infections in children [7]. Despite these antiviral properties, the clinical studies have not addressed the mechanism underlying the beneficial effects of OM-85. Most studies focussed on the regulation of immune cells including T-lymphocytes and natural killer T-cells, as well as on the synthesis of immunoglobulins [6]. In view of the current SARS-CoV-2 pandemics, the assessment of immunomodulators as a preventive therapy is of high interest. The findings of this study suggest that the antiviral effect of immunomodulators might result from the combination of various mechanisms instead of a single one.

Bacterial lysates such as OM-85 and Pulmonarom both originate from 5 different bacterial genera and are a mixture of bacterial proteins, lipid polysaccharides, and short-chain fatty acids. However, due to the different manufacturing processes, the nature of the components might be different. Thus, each of the two bacterial lysates will consist of different microbial-associated molecular patterns (MAMPs), which will target different pattern recognition receptors (PRRs). This might explain the observed different antiviral effects and innate immune response. For OM-85, its antimicrobial immune responses might be somehow compared similarly to the “Farm Effect” known from asthma patients being exposed to a variety of farm microbial components [26].

MAMPs induce the host defence through pattern recognition receptors such as TLRs. OM-85 has been reported to act through TLR4 and TLR2, thereby activating NF*κ*B and mitogen-activated protein kinases (MAPK) [27]. Furthermore, OM-85 has been reported to activate Erk1/2 MAPK, which resulted in the upregulation of *β*-defensin and the downregulation of ICAM1 [19]. The latter effects will increase the host defence against viruses through *β*-defensin and at the same time reduce the biding of specific viruses to the host epithelial cells. In the same study, OM-85 upregulated the expression of IFN-*β* secretion by human bronchial epithelial cells. Here, we show that in BMDCs, OM-85, but not Pulmonarom, upregulated type I IFN-*β*, a key element of host defence against viral infections. Future investigations are needed to assess details on the immune-regulation and importance of IFNs in the antiviral effects of OM-85. Our results suggest that blocking OM-85-induced IFN secretion in animal models of respiratory tract infection or in in vitro studies will be required to prove their role in the antiviral effect of this bacterial lysate.

The major group of human pathogenic RV includes about 90% of the 100 serotyped strains and uses ICAM-1 as the main receptor to bind and infect host cells [28, 29]. ICAM-1 is a molecule expressed on the surface of bronchial epithelial cells [30, 31]. In vitro, ICAM-1 also mediated the transmigration of polymorphonuclear leukocytes across airway endothelial and epithelial monolayers and their activation [32, 33]. The observation that both OBLs decreased RV-16-induced ICAM-1 expression might be interpreted as a protective function against viral infection. This idea was supported by the ability of OM-85 to increase the survival of RV-16-infected BEC. BECs activate the innate immune response via recognition of viral proteins by pattern recognition receptors (PRRs) including TLRs. TLR2 recognizes viral capsid proteins, whilst TLR7/8 sense viral nucleic acids present in the endosomal compartment [17, 34, 35].

Recognition of RV by PRRs causes the secretion of inflammatory cytokines, including IFN-*α* and IFN-*β*, the first line of defence against viruses [17, 34–36]. In response to viral infections, BECs also produce and release microbicidal *β*-defensins that possess a wide range of functions in regulating both innate and adaptive immunity [37]. In this study and in contrast to Pulmonarom, OM-85 increased the expression of IFN-*β* in BECs and DCs suggesting that increased IFN-*β* release might be an important antiviral mediator induced by OM-85.

The possibility of increasing the antiviral defence of bronchial epithelial cells in patients prone to recurrent viral infections is an attractive therapeutic goal [13, 14, 38, 39]. In this context, it was reported that OM-85 can promote BEC defence functions in response to RV-16 through the activation of the Erk1/2 MAPK pathway, thereby reducing ICAM-1 surface molecule expression and stimulating the release of *β*-defensin and the virus interacting proteins C1q-R [19]. Importantly, pretreatment with OM-85 over 24 hours prior to RV infection improved cell survival of BEC obtained from controls and patients with asthma or COPD; thus, there was no disease-specific response to OM-85 [19].

Therefore, the beneficial effects of OM-85 might be the result of the specific mixture of immune active components from different respiratory relevant microorganisms. It might, therefore, be impossible to clearly define a single mechanism as being responsible for the beneficial effects of OM-85 against viral infections. However, this will not diminish its preventive effects in patients who are at risk for reoccurring respiratory tract infections.

## 5. Conclusion

Pulmonarom and OM-85 liquid forms are two similar OBLs obtained with the same bacterial genera with minor strain differences. To investigate how this minor change could potentially affect the antiviral response, we designed a comparative study using both liquid forms, compared some features of their induced cell response and antiviral properties on epithelial cells, and finally evaluated selected aspects on dendritic cells.

Considering the limitation of the study with regard to the absence of concentration for one of the OBLs (Pulmonarom), we used a large set of serial volume dilutions to enable us to interpret the results. The study demonstrated that both OBLs not only elicit antiviral effects but also exert distinct product-specific antiviral responses on human BEC and mouse BMDCs with stronger antiviral properties for OM-85 in the models used in the study. Considering that the same bacterial genera were used to generate both OBL products, this important difference in protective cell response may originate not only from the PAMP content of each OBL product but also from the process used to manufacture these products. Further experiments are currently ongoing to confirm this hypothesis.

## Data Availability

The original data can be made available upon request from the corresponding author.
